# Faith-based Community Engagement in HIV-Testing and Awareness of HIV Status in Southern, Rural, African American Communities

**DOI:** 10.1007/s40615-024-02122-w

**Published:** 2024-09-16

**Authors:** J. M. Wise, M. C. Kempf, C. Ott, A. P. Footman, C. Hardy, B. Y. Araya, C. Walker, C. Latham, R. Stockett, G. L. Daniels, M. Alexander, R. G. Lanzi

**Affiliations:** 1 Department of Family, Community and Health Systems, School of Nursing, University of Alabama at Birmingham (UAB), Birmingham, AL 35294 USA; 2 Department of Health Behavior, School of Public Health, UAB, Birmingham, AL 35294 USA; 3 Department of Epidemiology, School of Public Health, UAB, Birmingham, AL 35294 USA; 4 Division of Infectious Disease, Heersink School of Medicine, UAB, Birmingham, AL 35294 USA; 5 Department of Medicine, Heerskink School of Medicine, UAB, Birmingham, AL 35294 USA; 6 O’Neal Comprehensive Cancer Center, UAB, Birmingham, AL 35294 USA; 7 100 Black Men of America, Atlanta, USA; 8 Department of Journalism and Creative Media, University of Alabama, Birmingham, AL 35294 USA

**Keywords:** CPBR, HIV testing, Rural South, Community-institutional relations, Faith-based initiatives, AAs

## Abstract

The Deep South is the epicenter of the HIV-epidemic in the United States, with rural AAs bearing the greatest burden. Traditional efforts to improve testing efforts have been largely unsuccessful due to their failure to recognize and leverage the sociopolitical and cultural factors that affect the uptake of HIV-screening interventions at the community level. The purpose of this study was to gain a deeper understanding of the socio-cultural contexts impacting HIV-testing in the rural South, and to assess strategies to increase testing in rural, Southern communities. Focus groups (*n* = 8) and semi-structured interviews (*n* = 31) were conducted among community and faith-based leaders in Alabama and Mississippi, to inform our understanding of local perceptions of HIV infection, barriers and facilitators impacting HIV-testing, and best strategies for improving testing efforts at the local level. Interviews and focus groups were audio-recorded, transcribed verbatim, and analyzed to extract major themes. While both faith-based and community leaders reported at least some stigmatizing attitudes towards HIV infection, faith-based leaders were more likely to report discomfort being around someone with HIV and were more likely to link the spread of HIV to immoral behaviors. The combination of the cultural importance of the Church, deep-seated religiosity among community members, and faith-based messages associating HIV infection with immorality directly impacted HIV stigma within the community-in turn, decreasing willingness to participate in HIV-testing, disclose positive HIV serostatus, or openly discuss transmission protection behaviors. The Church was identified as crucial to include to improve HIV-testing efforts in the rural South, due to their prominent sociopolitical roles within communities and ability to influence community members’ perceptions of HIV stigma. Faith-based leaderships should be included in initiatives to increase improve HIV-testing and awareness of status and reduce HIV disparities in the Deep South.

## Introduction

The South is the epicenter of the HIV epidemic in the United States (U.S.) [1], with geographic and socioeconomic disparities impacting HIV diagnosis, linkage to care, and mortality rates [1–3]. Southern suburban and rural areas are particularly burdened by the HIV epidemic, which may be partially due to a greater prominence of intersectional stigmas (i.e., homophobia, transphobia, and racism), which may further compound negative beliefs about HIV infection and hinder awareness and access to care [1, 2, 4, 5]. In particular, cultural and spiritual values within rural African American (AA) communities may negatively influence willingness to test, disclose ones HIV status, and access prevention and treatment services [3, 6–8]. While AA churches vary in their interpretation of the Bible and worship styles, well-intended pulpit messages related to the transmission of HIV (i.e., promiscuity, homosexuality, and shame) may increase negative beliefs, and perpetuate community stigma towards HIV infection among AA church members [9–13]. Moreover, the effects of these overlapping stigmas may be compounded by underlying forms of racism and discrimination, still present in the South [3, 14, 15]. Consequently, AA in the South are disproportionately burdened by the HIV epidemic [6]. While AAs represent just 13% of the U.S. population, they account for 54% of new diagnoses in the South and 43% nationwide [1, 16]. While interventions to alleviate these disparities have largely focused on increasing HIV-related knowledge, and access to testing and treatment services [1], evidence suggests that the most efficacious interventions also recognize the unique sociocultural environment in which vulnerable communities live in [17]. Therefore, the purpose of this study was to gain an in-depth understanding of the AA community perspective on HIV-testing and awareness in their communities, using in-depth interviews and focus group discussions with community stakeholders, including faith-based leaders, best equipped to provide an intimate knowledge of their communities.

## Methods

The current study occurred in three phases between 2014 and 2017 using a sequential transformative design to guide a deeper understanding of the socioeconomic and cultural factors influencing HIV-testing and awareness of status in the rural South [18]. The purpose of Phase I was to establish a baseline understanding of the factors that influence HIV-testing efforts in the Deep South. Participants (*n* = 62) were recruited from a convenience sample of volunteer lay-health advisors who had previously participated in a successful community-participatory initiative to improve breast cancer screenings in the rural South [19, 20]. Although we acknowledge that cancer stigma is different from HIV stigma, Phase I participants were important to include as they possessed a working knowledge gained from implementing screening efforts, and navigating socioeconomic and cultural barriers related to a stigmatized health condition in the South. Focus groups (*n* = 6) provided us the opportunity to understand what attitudes, beliefs, and social pathways had helped or hindered the uptake of community-based interventions to improve health screenings in the past [21]. Based on feedback from Phase I participants, Phase II plans were developed to include a broader section of community stakeholders across six counties in the rural South. Phase II interviews (*n* = 31) provided us opportunity to ask local community stakeholders (i.e., faith-based leaders, social workers, educators, and healthcare workers) what they believed to be the most important local factors impacting HIV-testing in the rural South. Phase II participants emphasized that although socioeconomic disparities still served as barriers to testing, much of the burden related to HIV-testing was related to the emotional burden of HIV stigma, and this stigma was heavily driven by the AA church. Therefore, Phase III of this study was developed to gain a better understanding of the dynamics between the AA church and community, and the relationships between faith-based messages, HIV stigma, and HIV testing in the rural South. Community stakeholders (i.e., teachers, community coaches, social workers, and health care workers) (*n* = 8) and faith-based leaders (*n* = 10) were purposefully recruited for focus groups (*n* = 2) from a single, predominantly AA, rural town in Mississippi [18]. A brief quantitative instrument was administered to better inform understanding of the types of attitudes and beliefs which influenced HIV testing in the South (see Table 1). This study was approved by the University of Alabama at Birmingham’s Institutional Review Board. All participants provided informed consent prior to focus group or interview participation. Descriptive statistics were computed for demographic and stigma data. Focus groups and interviews were audio-recorded, transcribed verbatim, and analyzed by two coders (C.O. and J.W.) using thematic analysis, as facilitated by NVivo12.

**Table 1:** Dimensions of HIV stigma among community stakeholders and faith-based leaders

Dimension of stigma	Community stakeholders Average score	Faith-based leaders Average score	Combined scores Average score
Concerns about occasional encounters (e.g., being around someone with HIV does not bother me)	3.46	3.00	3.20
Concerns about close encounters (e.g., I could not be friends or hug someone who has known HIV)	3.46	2.67	3.19
Responsibility and blame (e.g., my support for someone living with HIV depends on how the person was infected)	3.45	2.83	3.35
Liberalism (e.g., the spread of HIV is linked to the decline of moral values)	2.87	2.25	2.53
Non-discrimination (e.g., doctors who are infected with HIV should be allowed to go on working with their patients)	3.00	2.40	2.85
Confidentiality of status (e.g., I have the right to know if someone around me is infected with HIV)	2.96	2.00	2.74
Criminalization of transmission (e.g., transmitting HIV should be punishable by law.)	2.00	2.00	2.14
Overall Score	3.07	2.76	2.90

^*^Higher scores indicate less stigma

## Results

This study was a sequential, transformative study, in which the results of each phase guided the methods selected for subsequent phases, as well as the overall interpretation of study results. Focus group and interview objectives were similar, and included questions such as: *How do you think your community perceives HIV testing? What barriers do people in your community have with seeking HIV screening?* and *What suggestions do you have for reaching your community?*

### Phase 1

Phase I participants (*n* = 62) were mostly female (95.1%), AA (98.3%), who were 50 years of age or older (87.5%), married or widowed (50%), and college-educated (78.4%) [18]. All had previous experience implementing community-based initiatives to improve health screenings in the rural South, and focus groups were designed to gain a better understanding of the contexts participants believed would influence the successful implementation of an HIV-screening initiative among similar demographics. Participants described how deeply ingrained myths and misunderstandings surrounding HIV infection influenced fear and stigma within communities, and contributed to the false belief that only individuals engaging in stigmatized, high-risk behaviors (e.g., promiscuous sexual activity, IV drug use) needed to be tested. Widespread stigmatization of the behaviors associated with HIV transmission impacted willingness to test, and to disclose status, related to fear of social repercussion. Stigma was described so severely that it impacted willingness for community members to even discuss HIV-related matters, and would serve as a serious barrier to improving accurate information about HIV, and reducing negative connotations. Participants emphasized the critical nature of a thorough understanding of the inner workings and networks within community to identifying the source of misperceptions and stigmatization within communities, correcting long-held misperceptions surrounding HIV, and providing an opportunity to engage key community stakeholders who could facilitate the success of an HIV-testing initiative from within. Although stigma was emphasized as the leading barrier impacting HIV-testing, participants discussed that socioeconomic barriers continued to exist in the South (i.e., poverty, transportation, healthcare access), influencing testing (Table 2 portrays exemplar quotes from each phase).

**Table 2:** Barriers and solutions to HIV-testing and awareness of status in the Southern US

Barriers	Solutions
Faith-based messages promote stigma	Education alleviates stigma
• *When …I got hired with AIDS XXXX, my focus was reachin’ to churches. I had so much resistance from the ministers* • *We got more of us black people dyin’ because we [the church] don’t wanna talk about what’s really goin’ on* • *It’s a homosexual disease. It’s a intravenous drug use. It’s still that stigma on it* • *They think because you talk about it, you’re condonin’ it* • *It’s meant out of good, but it didn’t stop anything, and it made people just go underground*	• *If you reach out to all the pastors in this city and get them to come to a meeting….have the foundations, the funders, the health department heads…educate the pastors and tell them what you really wanna try to accomplish. That would be the biggest starting point as far as changing and getting a grip on this epidemic* • *Everyone need to be educated as to what this disease is, how it’s transmitted, how we can help one another, and especially build up people so that it won’t be spread* • *The more people know about it the less afraid they're gonna be, and therefore, the less stigma there will be. It's a cycle that we are constantly battling, but I think education is the answer to the question*
Socioeconomic disparities hinder access to testing	Partnering with the AA Church to increase access
• *As long as there’s poverty and people can’t get to you…* • *There are some people out there who probably would like to know, but finding easy access to the screening and it being economical…* • *You still have generations that are distrustful about their healthcare community just simply because of decades of discrimination or non-access*	• *In the African American community, …the church itself is what took care of the community* • *Part of that is providing not just the education, but the resources … creating health screenings where they’re accessible* • *In my profession, being a minister as well in healthcare as well—it seems like the models are goin’ towards networking with established providers where you can form collaborations and partnerships as well as to engage communities* • *Maybe somethin’ that you can have part of the church on a Sunday …there’s a lot of organizations that’ll…come in and…do health screenings for you* • *Break it down into smaller groups where people would feel more comfortable or even volunteering for testing*

### Phase II

For Phase II, we invited community stakeholders (*n* = 31) across six counties in Alabama and Mississippi to be a part of qualitative interviews designed to improve our understanding of the local contexts influencing HIV-testing within local communities in the South. Phase II participants included medical professionals (*n* = 6), peer educators (*n* = 7), church ministers (*n* = 6), youth advocates (*n* = 6), and county health department representatives (*n* = 6). Demographically participants were female (56.7%), AA (93.3%), 50 years of age or older (60%), married or widowed (58.6%), and college-educated (82.7%) [18]. Similar to Phase I participants, community stakeholders participating in Phase II described that although socioeconomic barriers existed in the South, widespread stigma and medical mistrust acted as the largest barriers influencing HIV-testing. HIV stigma permeated local communities, influencing the emotional burden (e.g., fear and shame) associated with testing, and negatively impacting community member’s willingness to engage in open discussion related to HIV. These negative beliefs would compromise the success of an HIV-screening initiative if not addressed from within. Yet, participants also described a potential solution to alleviate these barriers—the AA Church. Participants described how churches in the rural South often served as the cornerstone of AA communities, and that faith-based leaders had the capacity to influence community members, and correct misperceptions surrounding HIV infection. Moreover, churches had the knowledge and social standing to form partnerships with other local entities, which could make HIV education and testing services more accessible to community members.

### Phase III

To expand our understanding and perspective related to how community stakeholders and faith-based leaders could influence HIV-testing in the South, we invited community stakeholders (i.e., social workers, teachers, coaches, and healthcare workers) and local ministers to participate in separate focus groups in a single AA community in rural Mississippi. Phase III participants were male (66.7%), AA (100%), 50 years of age or older (66.6%), married or widowed (77.8%), and college-educated (94.4%). All were residents of the rural town where focus groups were held, with a vested interest in improving HIV-testing in their community. Phase III participants described that faith and the AA Church played a daily, guiding role in the lives of community members. Both community stakeholders and faith-based leaders agreed that HIV stigma present among community members could be traced back to the moral and religious beliefs valued within the Church. While participants believed the Church intended to promote messages associated with the moral beliefs of Christianity (e.g., abstinence before marriage and the avoidance of adultery), these messages were often harsh and hypercritical. Participants described how faith-based leaders were reluctant to emphasize factual, fear-negating truths associated with HIV, for fear of promoting immoral behavior. Devout followers then internalized the fear, shame, and embarrassment faith-based leaders attached to these behaviors, with the unintended consequence of discouraging willingness to test, and promoting stigmatization surrounding HIV.

Although participants agreed that many of the negative attitudes and beliefs surrounding HIV infection stemmed from well-intended faith-based messages, they also described the benefit of leveraging the Church, as the cornerstone of the AA community, to create change. Participants emphasized that faith-based leaders were well-respected in communities, ingrained into their existing social networks, and had the capacity and opportunity, to reach community members and shift the message surrounding HIV infection from one of stigma to one more focused on health. If faith-based leaders understood the impact of their morality focused, yet fear producing messages, they could strategically alter the messages to more positively influence the attitudes and beliefs surrounding HIV infection. Getting buy-in from the Church would be critical to re-educating community members, reducing long-standing negative attitudes and beliefs impacting HIV-testing, and improving HIV-testing in the rural South. Yet, faith-based were often not perceptive of how their faith-based messages impacted the HIV epidemic in their own community, and frequently held stigmatizing attitudes towards HIV, influencing their willingness to change faith-based messages (Table 1). Discussions emerged on the best tactics to reach faith-based leaders, and participants emphasized a logical, informational approach focused on personal and community health impact, would be key to promoting change.

With education and buy-in, the Church could not only be helpful in changing the culture surrounding HIV, but act as a community partner to facilitate the success of HIV-testing interventions. Churches represented a trusted, social platform, with community connections, and a charitable mission, which placed them in a prime position to increase the acceptability and feasibility of HIV-testing in the rural South. Through their infrastructure and social standing, the Church would be able to offer convenient, accessible testing through a trusted establishment, which would alleviate existing social (e.g., historical medical mistrust) and logistical (e.g., poverty, transportation, and access to medical care) barriers to testing (Table 2).

## Discussion

**Fig. 1:**
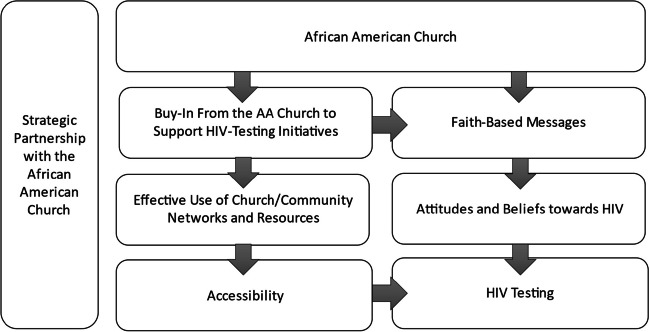
Strategic partnership with the African American church to increase access to testing and awareness of status

This study found that long-standing myths and inaccuracies continue to serve as a barrier to HIV-testing in the South, and that much of the moral and social stigma associated with HIV were also associated with faith-based messages, intended to encourage behavioral alignment with the social and religious values of the Church. Overwhelmingly, participants expressed how important it was to include the Church in efforts to change the culture surrounding HIV, due to their prominent social influence among communities, and deeply ingrained personal values (i.e., religious, social) held by most community members in the South. Through this engagement with faith-based leadership, community values and the social position of faith-based leadership could be leveraged to improve attitudes and knowledge surrounding HIV (see Fig. 1).

The results are aligned with the greater body of evidence suggesting that faith and religion heavily influence the HIV epidemic in the South [10, 12, 22]. AAs are more likely to report alliance with a particular religious denomination, attend regular church services, and consider religion an important part of everyday life compared to other races [23]. Further, the AA Church has long served as an essential part of AA communities in the South, often acting as a moral compass and social glue among communities [10, 12, 13]. Their involvement has frequently extended past the scope of religious function, as faith-based leaders have often engaged in political and civic advocacy efforts, impacting social and political causes in the South [10, 12, 13]. With this in mind, the results of this study corroborate existing evidence, and indicate that AA faith-based leadership has the social capacity to influence social and health-based change amongst communities in the South [3, 10, 12–14, 23].

The results of this study also corroborate the current evidence that in spite of the potential positive impact the AA Church to reach populations at risk for HIV in the South, religious attitudes and beliefs, stigmatizing behaviors associated with HIV transmission, were often associated with sexual immorality by the Church, and thus, continued to negatively influence community beliefs related to HIV infection in the South [3, 14, 23]. Notably, community beliefs and attitudes surrounding HIV, are not just driven by lack of knowledge surrounding HIV, but the social implications and culture created within communities [24, 25]. Due to the position of the AA Church as a cornerstone of the AA community, these social and cultural implications created through the Church are heavily ingrained into the overall function of the community [10, 12, 13]. Thus, the results of our study, and the evidence presented in the existing literature, implies that changing the culture within religious AA communities in the South must include a culture shift from within the Church. Evidence suggests that faith-based education efforts are effective in improving negative attitudes and beliefs (e.g., stigma) among both faith-based leaders and congregation members [22, 24, 26]. Because HIV-related stigma is not solely driven by lack of knowledge, both informational and socially equalizing components have been suggested as interventions to improve attitudes and beliefs towards HIV. For example, evidence suggests that both written information and brief workshops, targeted to enhance understanding of personal risk, matters of confidentiality, and procedures associated with HIV-testing and treatment services, are effective in increasing accurate knowledge surrounding HIV [24, 27]. Whereas, social components, such as providing exposure to individuals of similar social status, who are living with HIV, has been supported to increase empathy, respect, and promote conversation surrounding HIV [22, 25, 28]. Thus, both informational and socially equalizing components should be considered as interventions to reduce stigma among faith-based leaders and reduce stigma among AA rural communities in the South.

Finally, the results of this study corroborate the evidence that interventions designed to alleviate local disparities must consider the sociopolitical framework under which communities’ function [12, 29, 30]. Without this knowledge and consideration, interventions will consistently be unable to leverage existing networks, and the fundamental causes of disparities will be inadequately addressed, impacting the health and wellbeing of community members [23, 28, 29]. Due to the historical position of the AA Church among rural communities in the South, faith-based leaders must be included in conversations to identify local resources, and address local disparities in the rural South.

## Limitations

Limitations of this study include that geographic sampling for this study was limited to counties across Alabama and Mississippi. Although evidence suggests that there are common contexts across the rural South influencing the attitudes and beliefs surrounding HIV, we acknowledge there may be differences in sociopolitical and cultural beliefs across other communities in the South, which may impact the uptake of HIV-testing interventions in other ways. However, this research is beneficial in providing rich description of how the faith-based leaders influence community stigma towards HIV in the South, and provides the framework for recognizing local sociopolitical considerations across any Southern town. Additionally, political and denominational considerations were not collected as part of this study, which prevents the examination of how these contexts influence the attitudes, beliefs, and intensity of influence associated with HIV-testing in the South. However, the attitudes and influence of social, civic, and faith-based leaders were consistently captured across varying demographics and geographies throughout this study.

## Conclusion

Sociopolitical and cultural contexts continue to influence the HIV epidemic in the rural South. Initiatives focusing only on addressing the traditional disparities associated with HIV (i.e., poverty and inadequate access to healthcare) will likely miss key factors within communities serving as fundamental causes to the attitudes, beliefs, and behaviors associated with HIV-testing, thus, impacting community outcomes. Existing organizations, such as the AA Church, with long-standing social power and leadership within communities must be included in the conversation as stakeholders within their community, as well as for their capacity to generate social change. Through this inclusion, and additional respect given to the infrastructure of local communities, we can more effectively understand barriers, and leverage resources needed to create change.

## References

[1] Prevention CfDCa. HIV in the Southern United States. Updated May, 2016. 2018. https://www.cdc.gov/hiv/pdf/policies/cdc-hiv-in-the-south-issue-brief.pdf. Accessed 18 Jan 2018

[2] Pellowski J. Barriers to care for rural people living with HIV: a review of domestic research and health care models. J Assoc Nurses AIDS Care. 2013;24(5):422–37. 10.1016/j.jana.2012.08.007. 23352771 10.1016/j.jana.2012.08.007 PMC3640620

[3] Aholou TM, Cooks E, Murray A, et al. “Wake up! HIV is at your door”: AA faith leaders in the rural South and HIV perceptions: a qualitative analysis. J Relig Health. 2016;55(6):1968–79. 10.1007/s10943-016-0193-z. 26883229 10.1007/s10943-016-0193-z

[4] Darrow WW, Montanea JE, Fernandez PB, Zucker UF, Stephens DP, Gladwin H. Eliminating disparities in HIV disease: community mobilization to prevent HIV transmission among Black and Hispanic young adults in Broward County, Florida. Ethn Dis Summer. 2004;14(3 Suppl 1):S108–16. 15682779

[5] Perez-Brumer A, Nunn A, Hsiang E, et al. “We don’t treat your kind”: Assessing HIV health needs holistically among transgender people in Jackson, Mississippi. PLoS ONE. 2018;13(11):e0202389. 10.1371/journal.pone.0202389. 30383751 10.1371/journal.pone.0202389 PMC6211621

[6] Prevention CfDCa. HIV among AAs. 2018. https://www.cdc.gov/hiv/group/racialethnic/africanamericans/index.html. Accessed 18 Jan 2018

[7] Baral S, Logie CH, Grosso A, Wirtz AL, Beyrer C. Modified social ecological model: a tool to guide the assessment of the risks and risk contexts of HIV epidemics. BMC Public Health. 2013;13:482. 10.1186/1471-2458-13-482. 23679953 10.1186/1471-2458-13-482 PMC3674938

[8] Jeffries WI, Sutton MY, Eke AN. On the battlefield: the black church, public health, and the fight against HIV among AA gay and bisexual men. J Urban Health : Bull N Y Acad Med. 2017;94(3):384–98. 10.1007/s11524-017-0147-0. 10.1007/s11524-017-0147-0 PMC5481215 28409359

[9] Smith J, Simmons E, Mayer KH. HIV/AIDS and the Black Church: what are the barriers to prevention services? J Natl Med Assoc. 2005;97(12):1682. 16396060 PMC2640746

[10] Coleman JD, Tate AD, Gaddist B, White J. Social determinants of HIV-related stigma in faith-based organizations. Am J Public Health. 2016;106(3):492–6. 26794158 10.2105/AJPH.2015.302985 PMC4815751

[11] Cosentino LA, Campbell T, Jett A, et al. Use of nucleic acid amplification testing for diagnosis of anorectal sexually transmitted infections. J Clin Microbiol. 2012;50(6):2005–8. 10.1128/jcm.00185-12. 22493338 10.1128/JCM.00185-12 PMC3372150

[12] Griffith DM, Campbell B, Allen JO, Robinson KJ, Stewart SK. YOUR Blessed Health: an HIV-prevention program bridging faith and public health communities. Public Health Rep. 2010;125(1_suppl):4–11. 20408382 10.1177/00333549101250S102 PMC2788403

[13] MacMaster SA, Jones JL, Rasch RF, Crawford SL, Thompson S, Sanders EC. Evaluation of a faith-based culturally relevant program for AA substance users at risk for HIV in the southern United States. Res Soc Work Pract. 2007;17(2):229–38. 10.1093/hsw/32.2.151 17571650

[14] Stewart JM. A multi-level approach for promoting HIV testing within AA church settings. AIDS Patient Care STDS. 2015;29(2):69–76. 10.1089/apc.2014.0160. 25682887 10.1089/apc.2014.0160 PMC4322032

[15] Heckman BD. Psychosocial differences between whites and AAs living with HIV/AIDS in rural areas of 13 US states. J Rural Health. 2006;22(2):131–9. 10.1111/j.1748-0361.2006.00021.x. 16606424 10.1111/j.1748-0361.2006.00021.x

[16] Centers for disease control and prevention. *HIV Surveillance Report, 2017*. Vol. 29. 2018. https://www.cdc.gov/hiv/library/reports/hiv-surveillance.html. Accessed Mar 2019

[17] Wyatt GE, Williams JK, Gupta A, Malebranche D. Are cultural values and beliefs included in US based HIV interventions? Prev Med. 2012;55(5):362–70. 10.1016/j.ypmed.2011.08.021. 21884721 10.1016/j.ypmed.2011.08.021 PMC3736836

[18] Kempf MC, Ott C, Wise JM, et al. Universal screening for HIV and hepatitis C infection: a community-based pilot project. Am J Prev Med. 2018;55(5s1):S112-s121. 10.1016/j.amepre.2018.05.015. 30670196 10.1016/j.amepre.2018.05.015 PMC6548448

[19] Hardy CM, Wynn TA, Huckaby F, Lisovicz N, White-Johnson F. AA community health advisors trained as research partners: recruitment and training. Fam Commun Health. 2005;28(1):28–40. 10.1097/00003727-200501000-00006. 10.1097/00003727-200501000-00006 15625504

[20] Lisovicz N, Johnson RE, Higginbotham J, et al. The Deep South Network for cancer control. Building a community infrastructure to reduce cancer health disparities. Cancer. 2006;107(8 Suppl):1971–9. 10.1002/cncr.22151. 16921494 10.1002/cncr.22151

[21] Hardy CM, Wynn TA, Huckaby F, Lisovicz N, White-Johnson F. AA Community health advisors trained as research partners: recruitment and training. Fam Commun Health. 2005;28(1):41–50. 10.1097/00003727-200501000-00006 15625504

[22] Lanzi RG, Footman AP, Jackson E, et al. Love with No Exceptions: a statewide faith-based, university-community partnership for faith-based HIV training and assessment of needs in the Deep South. AIDS Behav. 2019;23:2936–45. 10.1007/s10461-019-02604-7. 31321638 10.1007/s10461-019-02604-7 PMC7075029

[23] Nunn A, Cornwall A, Chute N, et al. Keeping the faith: AA faith leaders’ perspectives and recommendations for reducing racial disparities in HIV/AIDS infection. PLoS ONE. 2012;7(5):e36172. 10.1371/journal.pone.0036172. 22615756 10.1371/journal.pone.0036172 PMC3353968

[24] Derose KP, Griffin BA, Kanouse DE, et al. Effects of a pilot church-based intervention to reduce HIV stigma and promote HIV testing among AAs and Latinos. AIDS Behav. 2016;20(8):1692–705. 10.1007/s10461-015-1280-y. 27000144 10.1007/s10461-015-1280-y PMC4945375

[25] Pettigrew TF, Tropp LR. A meta-analytic test of intergroup contact theory. J Pers Soc Psychol. 2006;90(5):751–83. 10.1037/0022-3514.90.5.751. 16737372 10.1037/0022-3514.90.5.751

[26] Berkley-Patton J, Bowe-Thompson C, Bradley-Ewing A, et al. Taking it to the pews: a CBPR-guided HIV awareness and screening project with black churches. AIDS Educ Prev. 2010;22(3):218–37. 20528130 10.1521/aeap.2010.22.3.218 PMC3924866

[27] Bradley ELP, Sutton MY, Cooks E, et al. Developing FAITHH: methods to develop a faith-based HIV stigma-reduction intervention in the Rural South. Health Promot Pract. 2018;19(5):730–40. 10.1177/1524839917754044. 29383967 10.1177/1524839917754044 PMC6211576

[28] Derose KP, Bogart LM, Kanouse DE, et al. An intervention to reduce HIV-related stigma in partnership with AA and Latino churches. AIDS Educ Prev. 2014;26(1):28–42. 10.1521/aeap.2014.26.1.28. 24450276 10.1521/aeap.2014.26.1.28 PMC3947594

[29] Corbie-Smith G, Adimora AA, Youmans S, et al. Project GRACE: a staged approach to development of a community-academic partnership to address HIV in rural AA communities. Health Promot Pract. 2011;12(2):293–302. 10.1177/1524839909348766. 20685913 10.1177/1524839909348766 PMC3063323

[30] Foster PP, Cooper K, Parton JM, Meeks JO. Assessment of HIV/AIDS prevention of rural AA Baptist leaders: implications for effective partnerships for capacity building in American communities. J Natl Med Assoc. 2011;103(4):323–31. 21805811 10.1016/s0027-9684(15)30313-8

